# Anodal Transcranial Direct Current Stimulation Enhances Survival and Integration of Dopaminergic Cell Transplants in a Rat Parkinson Model

**DOI:** 10.1523/ENEURO.0063-17.2017

**Published:** 2017-09-19

**Authors:** Christian Winkler, Janine Reis, Nadin Hoffmann, Anne-Kathrin Gellner, Christian Münkel, Marco Rocha Curado, Luciano Furlanetti, Joanna Garcia, Máté D. Döbrössy, Brita Fritsch

**Affiliations:** 1 Department of Neurology, University Hospital Freiburg, Freiburg, 79106, Germany; 2 Department of Neurology, Lindenbrunn Hospital, Coppenbrügge 31863, Germany; 3 Laboratory of Stereotaxy and Interventional Neuroscience, University Hospital Freiburg, Freiburg, 79106, Germany

**Keywords:** motor cortex, noninvasive brain stimulation, transcranial direct current stimulation

## Abstract

Restorative therapy concepts, such as cell based therapies aim to restitute impaired neurotransmission in neurodegenerative diseases. New strategies to enhance grafted cell survival and integration are still needed to improve functional recovery. Anodal direct current stimulation (DCS) promotes neuronal activity and secretion of the trophic factor BDNF in the motor cortex. Transcranial DCS applied to the motor cortex transiently improves motor symptoms in Parkinson’s disease (PD) patients. In this proof-of-concept study, we combine cell based therapy and noninvasive neuromodulation to assess whether neurotrophic support via transcranial DCS would enhance the restitution of striatal neurotransmission by fetal dopaminergic transplants in a rat Parkinson model. Transcranial DCS was applied daily for 20 min on 14 consecutive days following striatal transplantation of fetal ventral mesencephalic (fVM) cells derived from transgenic rat embryos ubiquitously expressing GFP. Anodal but not cathodal transcranial DCS significantly enhanced graft survival and dopaminergic reinnervation of the surrounding striatal tissue relative to sham stimulation. Behavioral recovery was more pronounced following anodal transcranial DCS, and behavioral effects correlated with the degree of striatal innervation. Our results suggest anodal transcranial DCS may help advance cell-based restorative therapies in neurodegenerative diseases. In particular, such an assistive approach may be beneficial for the already established cell transplantation therapy in PD.

## Significance Statement

Anodal direct current stimulation (DCS) is well established and widely used in experimental neuroscience and clinical studies to promote neuronal activity and learning. The underlying mechanisms include signaling of the trophic factor BDNF, including elevation of striatal BDNF in naïve rats. Here we demonstrate for the first time beneficial effects of anodal DCS on survival and integration of dopaminergic cell transplants in the 6-OHDA-lesioned striatum in a rat Parkinson model. Increased fiber outgrowth in the striatum was accompanied by pronounced improvement of pharmacologically induced motor behavior. Since anodal DCS is fully established and ready to use in clinical studies, this work provides a strong basis for future translational research in stem cell-based restorative therapy.

## Introduction

Transplanting dopamine-rich fetal ventral mesencephalic (fVM) cells into the striatum aims to reconstitute dopaminergic neurotransmission in Parkinson’s disease (PD; Winkler et al., 2005; Thompson and Björklund, 2012;Barker et al., 2015). This approach recently regained international attention and a phase 1 clinical trial is under way (Barker et al., 2015). To enable fast and optimal functional recovery, strategies to promote the survival of grafted dopaminergic cells and to accelerate their functional integration into the host neural circuitry are needed.

In contrast to physiologic brain development and function, cell-based therapeutic approaches create an experimental condition in which neurons at various developmental states and with differing subsets of active signaling pathways and cues interact to form a functional network. These are accompanied by common pathways that are independent of the developmental state. In early development of the nervous system, neuronal activity guides the integration of neurons into the neuronal network, neurite outgrowth, and axon guidance (Spitzer, 2006). Similarly, in the adult brain integration of newly born neurons, axonal sprouting after injury in the cortico-striatal system, and network reorganization by synaptogenesis are all subject to neuronal activity (Carmichael and Chesselet, 2002; Andreae and Burrone, 2014). This suggests that modulating neuronal activity can be adopted to promote integration of fetal mesencephalic cells into the adult striatal network. Other strategies to optimize the neurogenic environment include the local application of the neurotrophin BDNF. Evidence for its trophic effects on fetal cell survival and differentiation *in vitro* is undisputed (Hyman et al., 1991; Knüsel et al., 1991; Studer et al., 1995). *In vivo*, effects of BDNF on fiber outgrowth and behavior have also been promising (Sauer et al., 1993; Yurek et al., 1998, 1996), despite methodological challenges, including the route of administration due to the large molecule size (Yan et al., 1994; Krobert et al., 1997; Ratzka et al., 2012). Additionally, nonphysiologic continuous BDNF overexpression and trkB signaling may even have deleterious effects, such as seizures (Croll et al., 1999; Heinrich et al., 2011). Overall, optimizing the environment for fetal cell grafts by increased expression of BDNF in a physiologic range and overcoming the application obstacles hold promise for improving survival and integration of dopaminergic cell transplants.

Transcranial direct current stimulation (DCS) is a widely used noninvasive technique to modulate neuronal activity in humans and animal models (Bikson et al., 2016). Anodal DCS (anode placed above the region of interest) enhances neuronal activity (Bindman et al., 1962; Nitsche and Paulus, 2000). Motor cortical DCS promotes lasting behavioral improvements in human motor learning (Reis et al., 2009, 2015; Fritsch et al., 2010) and in neurologic disorders (Lefaucheur et al., 2016). These effects may be driven by BDNF-dependent synaptic plasticity as increased BDNF secretion and trkB receptor activation are essential for DCS-induced plasticity (Fritsch et al., 2010; Podda et al., 2016). Increases in neuronal activity and plasticity occur within a physiologic range (Fritsch et al., 2010), and without serious adverse events (Bikson et al., 2016).

Preliminary evidence from a limited number of studies suggests only minor and rather short-lived beneficial effects of anodal DCS on motor symptoms in PD patients and rodent models (Li et al., 2011; Elsner et al., 2016; Ferrucci et al., 2016). On the contrary, effects of DCS on restorative therapies, such as dopaminergic cell transplantation to the striatum, are largely unknown. DCS at high intensities had no detrimental effects on striatal transplants from a glia-like neural stem cell line, but network integration was not assessed (Keuters et al., 2015). The physiologic properties of DCS suggest its beneficial effects on the integration of fetal cell grafts into the adult striatal network.

Here, we tested both anodal and cathodal DCS in the 6-OHDA model treated with striatal fVM grafts, with the a priori hypothesis that only anodal DCS enhances graft survival, cell migration, and striatal dopaminergic reinnervation more than sham stimulation and thus promotes recovery of pharmacologically induced impairment of motor behavior.

## Materials and Methods

### Animals

Adult female Sprague Dawley rats (Charles River), weighing 200–225 g at the beginning of the experiment, were housed under a 12/12 h light/dark cycle with water and food *ad libitum*. All animal studies were performed according to the Animal Protection Law and Directive 2010/63/EU of the European Commission. Animal protocols were approved by the Commission for Animal Experimentation of the Regional Council of Freiburg and the Commission for Animal Experimentation of the University Medical Center.

### Striatal BDNF assessment post-DCS in naïve rats

Sixteen naive rats used for striatal BDNF protein measurements underwent surgery for transcranial DCS (description below) except for subcutaneous chest electrode placement, which was placed externally using a rubber chest electrode (20 × 15 mm) fitted into a vest for the single DCS session. Sham (0 A/m^2^, *n* = 7) or anodal (8 A/m^2^, *n* = 9) DCS was applied to the motor cortex once for 20 min in alert animals, and striata were dissected and shock frozen 1.5 h after the end of stimulation. Striata from individual animals were homogenized in extraction buffer (100 mM Tris/HCl, 1 M NaCl, 4 mM EDTA Na_2_, 2% Triton X-100, 0.01% sodium azide, and 1:100 protease inhibitor cocktail; Sigma-Aldrich). From the resulting supernatant, after 14,000 × *g* 10-min centrifugation at 4°C, the protein content per sample was determined by the bicinchoninic acid method (Pierce BCA protein assay kit; Thermo Scientific). The BDNF content in recovered protein samples was determined using a sandwich ELISA protocol by use of a mouse anti-human BDNF monoclonal capture antibody (1:100, R and D Systems catalog MAB648, RRID:AB_2064314). After washing, wells were incubated for 5 h at room temperature with blocking buffer (1% normal goat serum in TBS) followed by washing. Duplicate aliquots of tissue protein samples (in 1:10 in blocking buffer) or BDNF standards (0.8–125 pg/ml) were incubated overnight at 4°C. This was followed by washing and the addition of biotinylated mouse anti-human BDNF monoclonal detection antibody (1:500; R and D Systems catalog MAB648, RRID:AB_2064314) before incubation overnight at 4°C. After washing, streptavidin-β-galactosidase in blocking buffer (1:3000; Roche 11112481001) was added before incubation for 4 h at 4°C. Streptavidin-β-galactosidase activity was detected using 4-methylumbelliferyl-b-D-galactopyranoside (200 µM in K-phosphate buffer, 4°C overnight; Sigma-Aldrich). Fluorescence was detected by 365-nm excitation and 445-nm emission using a plate reader (Infinite M200; Tecan). BDNF content in striatal lysates was quantified within the range of the linear standard curve calculated from the known amount of the BDNF dilutions and normalized to the soluble protein that was detected in each sample (pg BDNF/µg protein). BDNF content of the striata from the stimulated hemisphere was normalized to the contralateral striatum to control for interindividual variance and then compared to sham interhemispheric ratios.

### Parkinson disease experiment

**Figure 1.**
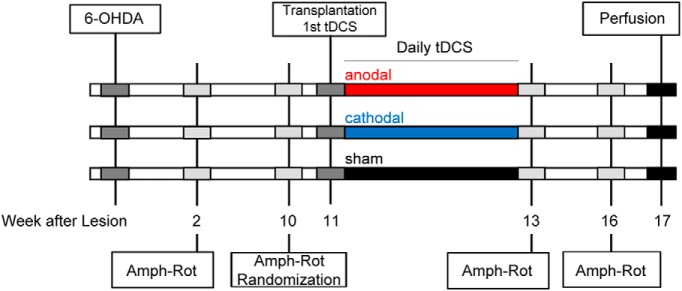
Experimental design. Animals were randomized to stimulation groups according to amphetamine rotation results (Amph-Rot) on week 10 after striatal lesion (6-OHDA). The daily 20-min stimulation started on the day of stem cell transplantation after recovery from anesthesia. Amphetamine rotation was retested immediately after the two-week stimulation period and for final assessment three weeks later and was followed by perfusion.

The time course of the experimental design is shown in Figure 1.

### 6-OHDA lesion

Animals received a unilateral 6-OHDA lesion by two stereotactic injections of 2.5- and 3-μl 6-OHDA (3.6 μg/μl) in 0.2% L-ascorbic acid-saline (Sigma-Aldrich) into the right medial forebrain bundle as previously described (García et al., 2011a): anteroposterior (AP) −4.4/−4.0 mm, mediolateral (ML) −1.2/−0.8 mm from bregma, and dorsoventral (DV) −7.8/−8.0 mm from dura, toothbar +3.4 mm. The unilateral dopaminergic denervation was behaviorally assessed preintervention by amphetamine-induced rotation at 2 and 10 weeks after 6-OHDA injection, and only animals performing more than four rotations were included in the study. Baseline rotation counts per group were 12.2 ± 1.3 (sham, *n* = 7), 13.0 ± 1.0 (anodal, *n* = 9), and 14.3 ± 1.5 (cathodal, *n* = 8), without significant differences between groups (*F*_(2,22)_ = 0.615, *p* = 0.55). Completeness of dopaminergic denervation following the 6-OHDA lesion was histologically confirmed postmortem by tyrosine-hydroxylase immunoreactivity (TH-ir) in the striatum.

### Preparation of the fVM cell suspension

Lewis wild-type female rats (Charles River) were time mated with Lewis GFP-transgenic heterozygote males ubiquitously expressing GFP under control of a cytomegalovirus/beta-actin promoter (Inoue et al., 2005) kindly provided by Eiji Kobayashi, Jichi Medical University, Japan. Single-cell suspension grafts were obtained from the VM from GFP-transgenic 14 d old (E14) Lewis rat embryos. In a previous study, dopamine cell suspensions obtained from E14 Lewis rats showed similar survival and behavioral improvements after intrastriatal transplantation into adult Sprague Dawley rats as compared to tissue obtained from E14 Sprague Dawley rats, even when immunosuppression was omitted (Krause et al., 2012). Dissection of the VM and preparation of single-cell suspensions from GFP-transgenic embryos were performed as described previously (Nikkhah et al., 1994; García et al., 2011b). For transplantation, cells were resuspended in Dulbecco´s modified eagle medium (DMEM; Gibco) containing 0.05% DNase (Sigma-Aldrich) at a concentration of 130,000 cells/μl and kept at room temperature until transplantation. After transplantation, leftover suspensions were used for estimation of cell viability using trypan blue exclusion; cell viability was still >95%.

### Cell transplantation and DCS electrode implantation

Animals underwent cell transplantation and electrode implantation during a single surgery session; performing surgeries in all rats on 1 d (∼16 h) as a multisurgeon team allowed us to use the same cell suspension for all animals. Surgery was performed using isoflurane anesthesia. To avoid order effects, animals from the different stimulation groups were enrolled in the surgery by stratified randomization (time of surgery, transplanter). First, the chest DCS electrode (platinum, 20 × 15 mm) was implanted with the connecting cable tunneled to the scalp. We then injected 1 µl of the cell suspension (∼130,000 cells) at a speed of 0.5 μl/min into the lesioned striatum, using a glass capillary (outer diameter 50–70 μm) fitted onto the needle of a 5-μl Hamilton syringe (coordinates from bregma: AP +0.2 mm, ML −3.5 mm, DV −4.5 mm, toothbar 0.0 mm), and the glass capillary was slowly retracted from the brain. Immediately thereafter, the 4-mm diameter cylinder for transcranial placement of the DCS electrode was fixed with acrylic cement to three screws on the skull (midpoint from bregma: AP +2 mm, ML −2 mm) together with the connector of the chest electrode. To avoid high-intensity current flow through the transplantation craniotomy, the borehole was sealed with cyanoacrylate glue and the rim of the cylinder was placed above the borehole.

### Transcranial DCS

Stimulation started on the implantation day, immediately after recovery from anesthesia (20 min after surgery) and was always performed in alert animals. Before transplantation the animals were allocated to anodal (anode above the motor cortex), cathodal (cathode above the motor cortex), or sham stimulation groups equally balanced with regard to performance during amphetamine rotation. DCS was applied through a 4-mm diameter sintered Ag/Cl electrode placed in the saline-filled cranial cylinder. Stimulation intensity was 8 A/m^2^ for anodal and cathodal DCS and 0 A/m^2^ for sham DCS. Stimulation duration was 20 min, resulting in a total charge of 0.96 C/cm^2^ per session. The stimulation intensity was far below the lesion threshold or threshold for activation of glia (Gellner et al., 2016). DCS was repeated once daily for a total of 14 consecutive days.

### Behavioral analysis

For this proof-of-concept study, motor behavior was analyzed as drug-induced rotational behavior in automated rotometer boxes for 90 min after injection of amphetamine (2.5 mg/kg, i.p.; Sigma-Aldrich). Data are expressed as total net full-body turns/min, with positive and negative values indicating rotations ipsilateral or contralateral to the lesion side, respectively. Amphetamine-induced rotation was performed at 2 and 10 weeks after the 6-OHDA lesion to estimate the extent of the lesion. Animals were included in the main experiment when they exhibited more than four full body turns per minute toward the side of DA depletion and were allocated to three equally performing groups for DCS. Assessment was performed at two and five weeks after transplantation to confirm the presence of surviving DA grafts and characterize the speed of behavioral recovery.

### Immunohistochemistry

Following the last behavioral test at five weeks after transplantation, brains were harvested after transcardial perfusion with 4% paraformaldehyde and cut at 40 μm using a freezing microtome. Free-floating sections (one in six series) were stained using a standard protocol (García et al., 2011a) with the primary antibodies mouse anti-TH (1:2500; Sigma-Aldrich catalog T1299, RRID:AB_477560) and mouse anti-GFP (1:500; Thermo Fisher Scientific catalog MA1-83783, RRID:AB_931093), using a biotinylated secondary antibody (anti-mouse IgG 1:200; Dako catalog Z0259, RRID:AB_2532147), avidin–biotin peroxidase solution (ABC Elite; Vector Laboratories) and 3,3’-diaminobenzidine (Merck) for visualization.

### Image analysis

In each animal, the GFP-positive graft core, the TH-ir total striatal area (both used for volume calculation), and density of the TH-ir fibers were assessed using ImageJ (National Institutes of Health). TH-ir cells in the striatum were counted using the Stereoinvestigator software (Microbrightfield; Stereo Investigator, RRID:SCR_002526), and the total number of cells was estimated using the Abercrombie formula (Abercrombie, 1946).

The GFP-positive graft core area was outlined manually and measured in all sections. The lesioned striatum was manually segmented for TH-ir fiber analysis, excluding the graft core and artifacts. Each individual image was normalized using the optical density (OD) of the TH-negative corpus callosum as a reference. The number of pixels above threshold was then calculated as the total area of TH-ir per section (5.16 µm/pixel). To calculate the volume, the graft core area and TH-ir fiber area were assumed to be circular. The interpolated volume between two sections was computed using the volume formula of a truncated cone [V = h*π/3 *(R^2^ + Rr + r^2^)], where h equates to the spacing between sections (40-µm sections + 120 µm intersectional = 160 µm) and R and r refer to the radius of the circular area of two consecutive sections (*n* and *n* + 1). The radii were derived from the circular area r = √(A/π). The final formula then reads as follows: V_n_ = (160/3* π)*[(A_n_/π) + √(A_n_/π)*√(A_n+1_/π) + (A_n+1_/π)]. All intersectional volumes were summed up to give the total graft core or fiber volume per animal.

In addition, TH-ir fiber density of the transplant was calculated in each section as the mean OD of TH-positive fibers and normalized to the mean OD of the contralateral unlesioned striatum, i.e., fiber density is given as a percentage. For this, the aforementioned thresholded and segmented area of TH-positive fibers was used as a mask applied to the original TH-stained section, and the mean gray value was assessed by ImageJ. The outlines of the thresholded lesioned striatum were then flipped horizontally and used to threshold and measure the mean gray value of the corresponding unlesioned striatal area with the threshold value derived from the unlesioned hemisphere. As total dopaminergic reinnervation is characterized by fiber volume and fiber density, one compound measure, the integrated fiber density, was calculated as the area of TH-ir fibers multiplied by the relative OD within the area. To assess a directed fiber outgrowth in relation to the DCS electrode, the dorsal and ventral portions of TH-ir pixels were obtained by setting a horizontal line through the center of the graft core and calculating a dorsal/ventral ratio.

### Statistical analysis

All statistical evaluations were performed using IBM SPSS version 20 (RRID:SCR_002865). The Kolmogorov–Smirnov test was used to test for normal distribution of the data. All animals fulfilling the criteria for inclusion (sufficient 6-OHDA lesion, enough rotations on the behavioral test) were included in the final data analysis (sham *n* = 7, anodal *n* = 9, cathodal *n* = 8 rats). Separate comparisons between sham and anodal DCS as well as sham and cathodal DCS were planned a priori. Hence, *t* tests for independent samples with factor stimulation as the grouping variable were performed for the striatal BDNF content (only sham vs anodal DCS), GFP graft core volume, TH-ir cell count, TH-ir cells per graft core volume, total volume and fiber density of the TH-ir fibers, and direction of fiber outgrowth (dorsal/ventral ratio) as the independent variables. Nonparametric Z-statistics (Mann-Whitney *U* test) was used when normal distribution of the data was not given. Regression analysis with TH-ir fiber volume as the dependent variable and TH-ir CELL COUNT and GROUP as coefficients was performed to assess the direct relation between fiber outgrowth and TH-ir cell count. Behavioral amphetamine-induced rotation data were analyzed by repeated measures ANOVA with factors TIME (baseline, two weeks, and five weeks after transplantation) and GROUP (sham/anodal in comparison 1, sham/cathodal in comparison 2) as the independent variables and behavioral performance in the two tests as the dependent variable. To assess dependence of behavioral recovery on the degree of reinnervation across all animals, we performed regression analysis with behavioral improvement achieved at week 5 after transplantation as the dependent variable and integrated fiber density and GROUP (sham, anodal, cathodal DCS) as coefficients. For all analyses, *p* < 0.05 was set as the level of significance. For the directed hypothesis of an increased striatal BDNF expression following DCS in naïve rats an upper tailed *t* test comparison was used; all comparisons in the PD experiment were performed as two-tailed tests. We did not correct for family-wise error rates as the comparison between stimulations is exploratory (procedure recommended in Bender and Lange, 2001).

## Results

### Effect of DCS on striatal BDNF protein content in naïve rats

**Figure 2.**
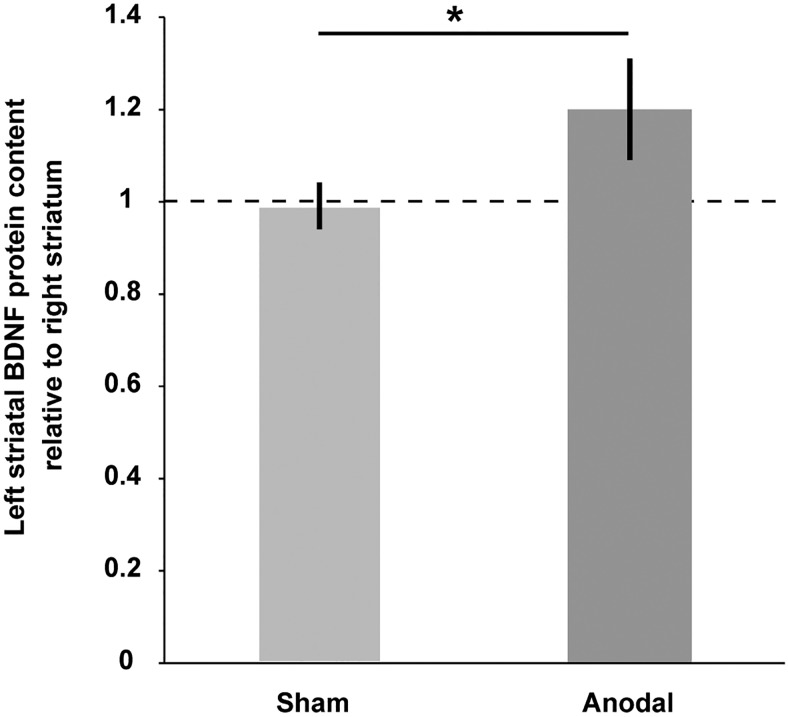
Anodal DCS applied to the left motor cortex enhanced ipsilateral striatal BDNF protein expression in naive rats. Left striatal BDNF protein content is normalized to the content of the contralateral nonstimulated striatum (1 = 100%) and compared between the two DCS stimulation conditions [sham DCS (*n* = 7) and anodal DCS (*n* = 9)]. Mean ± SEM; two-tailed Student’s *t* test; *p < 0.05.

1.5 hours after receiving 20 min of anodal or sham DCS over the left motor cortex, BDNF protein levels were increased by 20% in the striatum of the stimulated hemisphere relative to the contralateral hemisphere (120.1 ± 10.6%, *n* = 9); this increase was not observed after sham stimulation (99.1 ± 4.7% relative to the contralateral hemisphere, *n* = 7; *t*_(10.858)_ = −1.81, *p* = 0.049; Fig. 2).

### Anodal DCS enhances graft volume, TH-ir cell count, and dopaminergic reinnervation

**Figure 3.**
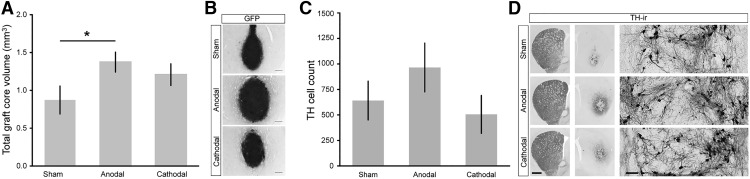
Differential effects of DCS on graft core volume and TH-ir cell count. ***A***, Anodal stimulation resulted in an increased transplant survival reflected in a significantly larger graft core volume compared to sham, which was not significant with cathodal DCS. ***B***, GFP graft cores are illustrated by representative micrographs from each DCS group. Scale bar: 250 µm. ***C***, The number of TH-ir cells in the anodal group was higher compared to the sham DCS group; no increase was present with cathodal DCS. ***D***, TH-ir cells as well as fiber outgrowth are illustrated by representative micrographs from each DCS group. TH-ir cells did not migrate out of the graft core. Scale bars: slice, both hemispheres 1 mm; high-magnification inset, 100 µm. Mean ± SEM; two-tailed Student’s *t* test each active stimulation versus sham; *p < 0.05.

Of the estimated initially grafted 13.000 dopaminergic cells (∼10% of 130,000 transplanted fetal cells) TH cell survival was ∼5% in sham stimulated rats, comparable to other transplantation studies using a similar design (García et al., 2011a, 2011b). Anodal DCS gave rise to better general transplant survival since animals in this group had a significantly larger GFP-positive graft core volume (1.37 ± 0.13 mm^3^, *n* = 9) than the sham DCS-treated group (0.87 ± 0.19 mm^3^, *n* = 7, *t*_(14)_ = −2.26, *p* = 0.040; Fig. 3*A*,*B*). In accordance, a higher count of TH-ir cells was found in the anodal DCS group (965 ± 239 cells, *n* = 9; Fig. 3*C*) compared to sham DCS (641 ± 190 cells, *n* = 7; (Z(14) = −1.0, p = 0.315). The amount of TH-ir cells per total graft core volume was not affected by anodal DCS (sham, *n* = 7, 835 ± 153 cells/mm³; anodal, *n* = 9, 645 ± 116 cells/mm³; t(14) = −1.01, p = 0.328). In all animals, TH-ir cells were within the dense graft core area; there was no cell migration to areas aside from the graft, neither with TH-staining nor with GFP (Fig. 3*D*). The majority of TH-ir cells settled at the border of the graft (Fig. 3*D*, inset).

**Figure 4.**
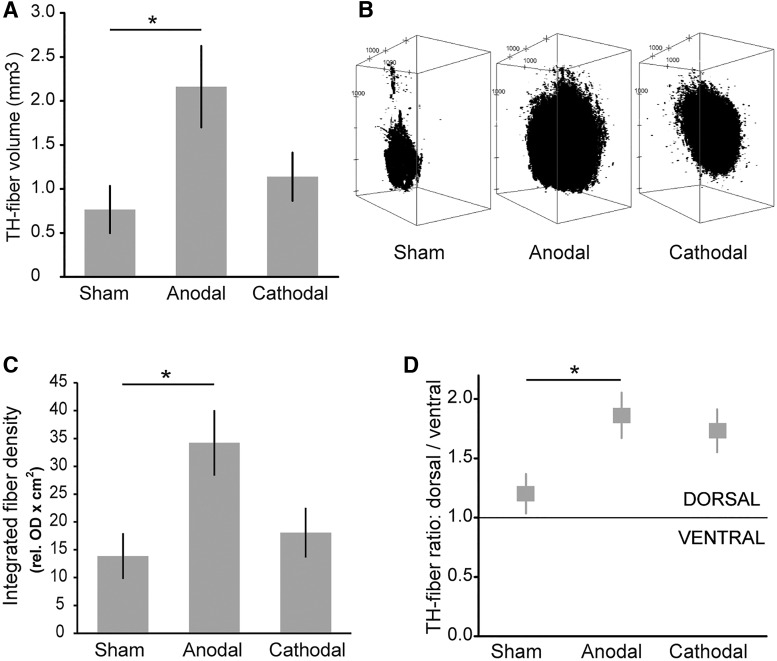
DCS exerted polarity-specific effects on fiber volume, density, and outgrowth direction. ***A***, Anodal DCS significantly enhanced the TH-ir fiber volume surrounding the graft. ***B***, Representative 3D reconstruction of TH-fiber volume in the three groups. Properties of binarized images stacks of individual fiber volumes were adapted to the original slice thickness (40 µm) and spacing between consecutive slices (120 µm). ImageJ plugin 3D viewer was used to render a 3D model of the stack. Stacks are rotated around the *y*-axis at an angle of 30°, i.e., volumes are shown from a diagonal perspective. Distance between axes tick marks is set to 1000 µm. Note that the fiber volume is largest in the anodal DCS condition. ***C***, Integrated fiber density, the relative OD of TH-ir fibers multiplied by the reinnervated area, was significantly higher in the anodal DCS group compared to sham DCS. ***D***, Both active stimulation paradigms resulted in a larger amount of TH-ir fiber outgrowth directed toward the cranial electrode (dorsal), with only anodal DCS reaching the level of significance. Mean ± SEM; two-tailed Student’s *t* test each active stimulation versus sham; *p < 0.05.

Fiber outgrowth, assessed as the total volume of the TH-ir fibers around the excluded graft core, was significantly increased in anodal DCS-treated animals (2.16 ± 0.44 mm³, *n* = 9) compared to sham DCS (0.77± 0.27 mm³, *n* = 7, *t*_(14)_ = −2.54, *p* = 0.024; Figs. 3*D*, 4*A*). The magnitude of dopaminergic reinnervation can be reflected by both the volume (Fig. 4*A*,*B*) and the density of TH-ir fibers relative to the unlesioned striatum. Within the reinnervated area, TH-ir fiber density relative to the fiber density of the unlesioned striatum was similar in the two groups (sham 64%, anodal 68%; *t* = −0.606, *p* = 0.554). Integration of both measures of graft-derived striatal dopaminergic reinnervation (fiber density × area) resulted in a significantly higher compound parameter (integrated fiber density) after anodal DCS (*n* = 9 vs sham DCS *n* = 7: *t*_(14)_ = −2.67, *p* = 0.018; Fig. 4*C*). As expected, across all animals, TH cell count was identified as a predictor of integrated fiber density in the regression analysis (b = 0.64, *t*_(13)_ = 4.19, *p* = 0.001). Hence, TH cell count explained a significant amount of variance of integrated fiber density (*F*_(1,13)_ = 16.57, *p* = 0.0003, *R* ^2^ = 0.718), indicating that survival of grafted dopaminergic cells is a prerequisite for subsequent fiber outgrowth. The anodal DCS group also showed more fiber outgrowth per TH-ir cell than the sham DCS group, hence stimulation type (GROUP) represented a general predictor of fiber outgrowth (b = 0.413, *t*_(13)_ = 2.71, *p* = 0.018). With regard to the direction of fiber outgrowth (Fig. 4*D*), sham-stimulated grafts represented a relatively homogeneous outgrowth, equally distributed between a dorsal (toward DCS electrode) and a ventral portion of the striatum from a virtual horizontal plane through the graft core (*n* = 7, dorsal/ventral ratio 1.2 ± 0.2). Anodal DC-stimulated grafts showed a greater proportion of fibers oriented dorsally (*n* = 9, dorsal/ventral ratio 1.9 ± 0.2; *t*_(14)_ = −2,52, *p* = 0.024).

### Cathodal DCS negatively affects TH-ir cell count within the graft core and does not affect dopaminergic reinnervation

Cathodal DCS did not significantly enhance general transplant survival, although animals numerically expressed larger graft core volumes (1.21 ± 0.14 mm^3^, *n* = 8) compared to the sham DCS group (0.87 ± 0.19 mm^3^, *n* = 7; *t*_(13)_ = −1.45, *p* = 0.171; Fig. 3*A*,*B*). TH-ir cell count was similar in the cathodal (505 ± 186 cells, *n* = 8) and sham DCS group (641 ± 190, *n* = 7; *t*_(13)_ = −0.51, *p* = 0.62; Fig. 3*C*). The amount of TH-ir cells per total graft core volume showed lower TH-ir cell density in the core of cathodal DCS-stimulated animals (416 ± 132 cells/mm³, *n* = 8; *t*_(13)_ = 2.08; *p* = 0.058) compared to sham-stimulated animals (835 ± 153 cells/mm³, *n* = 7). Furthermore, cathodal DCS (*n* = 8) did not affect TH-ir fiber outgrowth around the transplant (1.14 ± 0.29 mm³) and fiber density (64% of the unlesioned striatum) compared to sham (*n* = 7, volume: *t*_(13)_ = −0.94, *p* = 0.37; density: *t*_(13)_ = 0.1, *p* = 0.92; Fig. 4*A*,*B*). In accordance, integrated fiber density did not differ (*t*_(13)_ = −0.69, *p* = 0.51; Fig. 4*C*). Interestingly, the dorsal/ventral ratio of fiber outgrowth direction was affected by cathodal DCS tending toward a dorsal orientation (*n* = 8, dorsal/ventral ratio 1.7 ± 0.2; Fig. 4*D*); statistically this comparison did not reach significance compared to sham DCS (*t*_(13)_ = −2.02, *p* = 0.07, *n* = 7).

### Striatal reinnervation determines behavioral recovery

**Figure 5.**
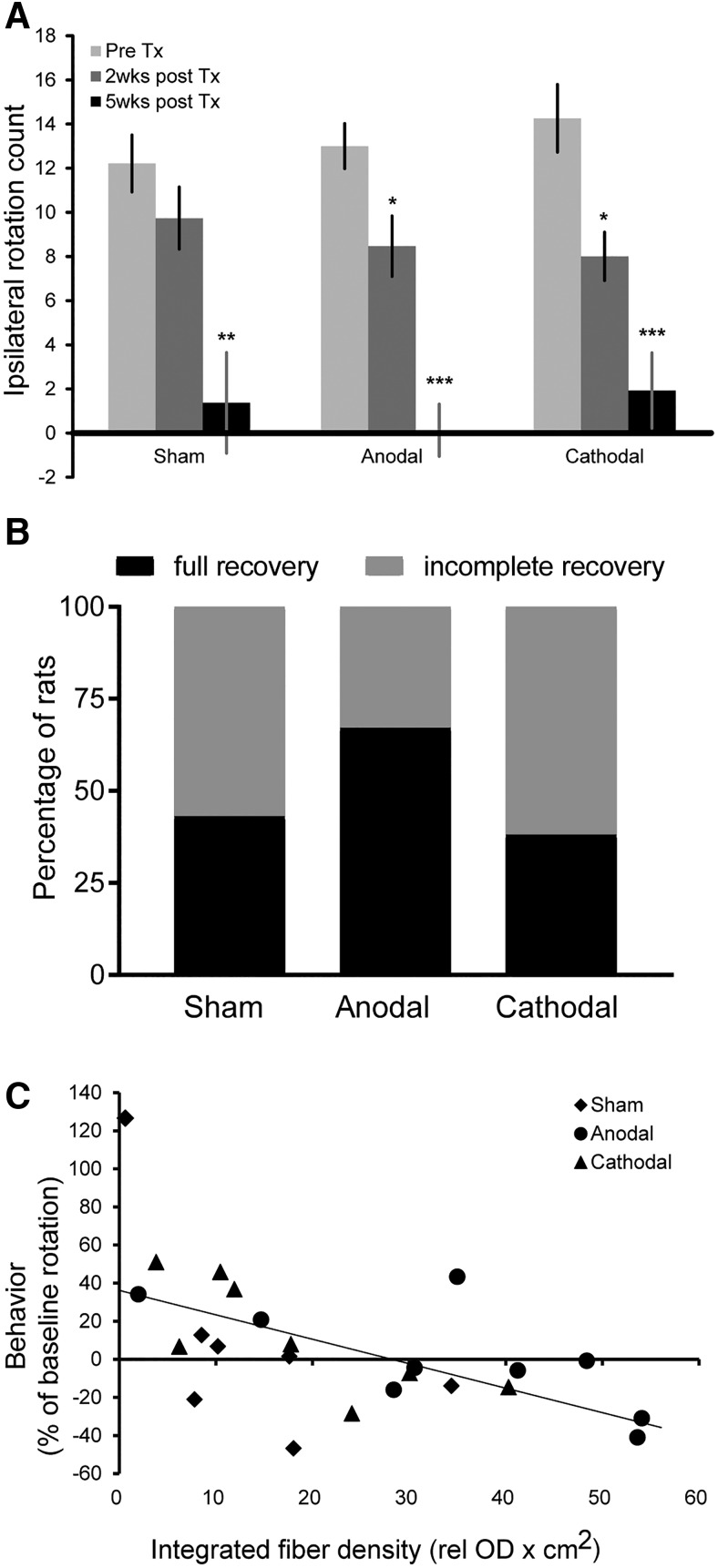
Behavioral recovery was more pronounced following anodal transcranial DCS, and behavioral effects correlated with the degree of striatal reinnervation. ***A***, Anodal and cathodal stimulation led to a significant decrease of amphetamine-induced ipsilateral rotation two weeks after transplantation; this effect was not seen with sham DCS. By five weeks, all groups showed significant resolution of ipsilateral rotations. ***B***, Animals in the anodal DCS presented the highest amount of animals with full behavioral recovery, measured as lack of ipsilateral rotation or even contralateral rotation after amphetamine application. ***C***, Generally, larger TH-fiber outgrowth was associated with improvement of baseline rotation five weeks after transplantation. Mean ± SEM. Repeated measure ANOVA, two- and five-week data in each group versus pretransplantation; *p < 0.05; **p < 0.01; ***p < 0.001.

Amphetamine-induced rotation was assessed pretransplantation and two and five weeks after transplantation/stimulation to behaviorally confirm the presence of dopaminergic reinnervation and to characterize the speed of behavioral recovery under the different DCS conditions (sham *n* = 7, anodal *n* = 9, cathodal *n* = 8). Compared to pretransplantation, animals showed a gradual reduction of ipsilateral rotations over time in all groups (Fig. 5*A*). For anodal versus sham DCS, the ANOVA revealed a significant effect of factor TIME (*F*_(2,23)_ = 61.435, *p* < 0.0001) but no effect of factor GROUP (*F*_(1,23)_ = 0.136, *p* = 0.72) and no GROUP × TIME interaction (*F*_(2,23)_ = 0.583, *p* = 0.57). A similar result was found for cathodal DCS compared to sham (factor TIME: *F*_(2,22)_ = 44.61, *p* < 0.0001, factor GROUP *F*_(1,22)_ = 0.03, *p* = 0.87) GROUP × TIME interaction: *F*_(1,22)_ = 1.18, *p* = 0.32). Group-wise *post hoc* testing for factor TIME indicated significant improvements from pretransplantation rotation counts in the two active stimulation groups (*p* = 0.04 for anodal and *p* = 0.02 for cathodal DCS) at week 2, which was not present in the sham group (*p* = 0.22), suggesting a smaller time constant of recovery under this condition. By week 5, all groups showed significant recovery compared to the pretransplantation condition (sham *p* = 0.005, anodal *p* < 0.0001, cathodal *p* = 0.0004; Fig. 5*A*). Overall recovery appeared more pronounced following anodal DCS since 67% of animals in this group showed no rotation or overcompensation while this was less frequent in the other groups (43% in the sham group and 38% in the cathodal group; Fig. 5*B*). Across all groups, the degree of graft-related striatal TH-ir innervation (integrated fiber density) was a significant predictor of the behavioral improvement achieved five weeks after transplantation (b = −0.58, *t*_(21)_ = −3.22, *p* = 0.004). Hence, striatal reinnervation explained a significant amount of variance of behavioral improvement (*F* = 5.199, *p* = 0.015, *R* ^2^ = 0.33; Fig. 5*C*). Group assignment per se was not a significant predictor in the model (b = 0.08, *t*_(21)_ = 0.46, *p* = 0.65).

## Discussion

This proof-of-concept study reports enhanced graft survival and dopaminergic network integration of grafted fVM cells following anodal DCS in a rodent model of PD likely by creating a trophic and neuroplastic milieu. Graft-mediated improvements in pharmacologically tested motor behavior are dependent on the degree of reinnervation.

Anodal DCS supports motor network plasticity across species. Factors contributing to this finding include increased neuronal activity (Bindman et al., 1962; Nitsche and Paulus, 2000), enhanced synaptic plasticity (Fritsch et al., 2010; Ranieri et al., 2012; Rohan et al., 2015; Podda et al., 2016), improved motor learning (Reis et al., 2009; Fritsch et al., 2010; Buch et al., 2017), and increased corticostriatal connectivity (Polanía et al., 2012). Moreover, DCS-related synaptic plasticity is mediated by increased BDNF signaling in the motor cortex (Fritsch et al., 2010), and in subcortical brain regions, e.g., the hippocampus (Podda et al., 2016). To support our assumption that anodal DCS applied to the primary motor cortex also exerts remote effects onto the ipsilateral striatum, potentially creating a neurogenic environment, we first confirmed enhanced ipsilateral striatal BDNF levels after anodal DCS in naïve rats.

While solely applied DCS has no beneficial effects on motor deficits in PD patients and animal models (Li et al., 2011; Elsner et al., 2016; Ferrucci et al., 2016), these benefits may be reached by enhanced striatal network integration of fVM cell transplants. In grafted rats, daily anodal DCS over the motor cortex significantly enhanced overall graft survival, i.e., volume, by a remarkable 56%. More specifically, this was reflected in a ∼50% higher number of TH-ir cells compared to sham stimulation. Transplant and TH-ir cell survival may be promoted by interventions that directly or indirectly enhance neuronal corticostriatal activity, connectivity and plasticity. Indeed, a STN lesion, known to enhance cortical input to the striatum, crucially enhanced survival of striatal fVM grafts in the 6-OHDA rat model (Cordeiro et al., 2014). In the same animal model, stimulation of the STN, exerting a striatal activation pattern similar to a STN lesion, increased the concentration of BDNF in the nigrostriatal system (Spieles-Engemann et al., 2011) and improved survival of dopaminergic cell transplants (Furlanetti et al., 2015). While the supportive effect of direct BDNF application on graft survival *in vitro* is widely accepted, *in vivo* data are currently inconclusive and the role of BDNF has not been directly investigated in the present *in vivo* experiment. Environmental enrichment also enhanced corticostriatal plasticity and graft survival (Mazzocchi-Jones et al., 2011), providing a technique to increase BDNF more physiologically in the intact and grafted striatum. Anodal DCS, STN lesion/stimulation, and enriched environment may share the physiologic enhancement of cortico-striatal neuronal activity and striatal BDNF as mechanisms underlying improved graft and TH-ir cell survival.

In our study, TH-ir cells per total graft core volume were similar with anodal and sham stimulation. This suggests that anodal DCS improved overall cell survival without specifically directing progenitor cells within the graft toward a dopaminergic phenotype. The grafted and stimulated cells (including neurons, stem cells, and glia) did not migrate beyond the graft core and the localization pattern resembles that of other studies with the same PD model but without electrical stimulation (Sauer et al., 1993; Cordeiro et al., 2014). At first sight, this finding might be surprising since galvanotactic effects of electrical fields have been reported for different brain cell types (Li et al., 2008; Yao et al., 2008; Arocena et al., 2010; Zhang et al., 2011). In contrast to our setting, these stimulations were applied much longer (hours) in a milieu not hindering spatial movement compared to tissue. Electrical fields resulted in only a few micrometers of cell movement per hour, most pronounced with extremely high-stimulation intensities. One *in vivo* study (Rueger et al., 2012) reported increased endogenous neural stem cell counts in the cortex under the electrode after motor cortical cathodal DCS. As the cells’ origin was not investigated local cell proliferation, as seen with other stimulation patterns (Jahanshahi et al., 2013), cannot be excluded. Accordingly, ten sessions of 143 A/m^2^ anodal DCS resulted in diffuse and undirected short distance migration adjacent to glia-like neural stem cell grafts (Keuters et al., 2015). Of note, these studies used DCS intensities associated with inflammatory responses (>15 times higher than our stimulation and 150 times higher than the human application). This is particularly important since acute inflammatory cascades after high-intensity stimulation may secondarily induce cell migration not directly dependent on DCS guidance, but, for example, on microglia activation (Belmadani et al., 2006). In our study, anodal and cathodal DCS were applied at 8 A/m^2^, clearly below the threshold for neurodegeneration and microglia activation (Liebetanz et al., 2009; Bikson et al., 2016; Gellner et al., 2016). Together, differences between studies with regard to cell migration can be explained by various stimulation patterns, intensities, and cell types. There was no observable migration of any cell type outside the fVM cell graft *in vivo* when subjected to brief, daily, low-intensity DCS for two weeks.

Anodal DCS significantly supported graft-derived striatal reinnervation, measured as the area and density of TH-ir fiber outgrowth, compared to sham stimulation. Previous studies have shown that up to a critical graft volume, larger surviving grafts lead to increased striatal reinnervation (Winkler et al., 1999; Kirik et al., 2001). Accordingly, we found that the higher number of TH-ir cells explained the higher dopaminergic fiber outgrowth. Moreover, anodal DCS increased TH-ir fiber outgrowth per grafted dopaminergic cell, bearing the potential to overcome the assumed fiber outgrowth limitations at critical graft volumes.

Besides the degree of striatal reinnervation, its anatomic location represents a critical factor for behavioral improvements (Mandel et al., 1990; Hagell and Brundin, 2001; Grealish et al., 2010). In the current study, anodal DCS led to directionality of TH-ir fiber outgrowth toward the dorsal striatum (i.e., toward the electrode). Directed fiber outgrowth can result from (1) a galvanotactic effect on outgrowth and orientation of fibers, independent of neuronal (network) activity and/or (2) an activity and guidance molecule (e.g., trophic factors) dependent effect on sprouting. From extensive work on isolated cultured neurons it is well known that the total neuronal outgrowth volume is increased and directed toward the cathode at field strengths of 7-1000 mV/mm (Jaffe and Poo, 1979; Patel and Poo, 1982; Bedlack et al., 1992; McCaig et al., 2000; Pelletier et al., 2015); <1 mV/mm estimated in humans (Datta et al., 2009). In the present study, TH-ir fiber outgrowth was most pronounced toward the anode. This finding suggests that *in vivo* outgrowth direction and network formation are not explained by galvanotaxis seen in cell culture. *In vivo* the direction of neuronal outgrowth may indicate the net effect of endogenous and exogenous electrical fields interacting with other directional signals, such as guidance molecule secretion and increased neuronal activity. A trend toward a dorsal fiber outgrowth bias was also seen for cathodal DCS. As decreased neuronal activity (Bindman et al., 1962) is expected with cathodal stimulation, the described galvanotactic effects on neurites toward the cathode might dominate under this condition.

Anodal DCS augmented the graft-induced restitution of motor function. Anodal DCS-stimulated rats showed the highest proportion of animals (67% vs 43% in the sham group) with complete restitution of amphetamine rotational behavior five weeks after transplantation. While cathodal stimulation also facilitated restitution of motor function after two weeks, there was no carryover of this effect to five weeks (38% of animals with normalized rotational behavior). It is well known that the magnitude of graft-related dopaminergic striatal reinnervation determines the functional outcome of PD patients, primates, and rodents (Mandel et al., 1990; Hagell and Brundin, 2001; Grealish et al., 2010). In line with these findings, TH-ir fiber outgrowth was a significant predictor for restitution of drug-induced rotation and was numerically highest with anodal DCS.

A trend toward enhanced graft core survival was seen with cathodal DCS but was not reflected in TH-ir cell survival. Cathodal DCS even led to a 50% reduced TH-ir cell count when normalized to the graft core volume. Given the dependency of fiber outgrowth on the cell count, cathodal DCS did also not enhance dopaminergic fiber outgrowth. Moreover, the lack of effect on striatal reinnervation explains the low normalization rate in drug-induced behavior five weeks after transplantation, altogether indicating a polarity and stimulation-specific beneficial effect of anodal DCS.

### Final remarks and limitations

The combined cell transplantation and DCS electrode implantation procedure as well as acute and consecutive two weeks of daily DCS were well tolerated without side effects. Graft vitality and striatal reinnervation was indicated by increased normalization of amphetamine-induced ipsilateral rotation over time and verified by *post hoc* histologic analysis. As expected for this rat model, graft-induced behavioral recovery is typically reached a few weeks after transplantation. To increase the likelihood of unraveling beneficial effects of DCS and to better dissect stimulation effects on structural and behavioral outcome parameters, we used a reduced graft size in only one location. Transplantation led to almost full recovery after five weeks in sham-stimulated animals leaving the effect of anodal DCS on graft survival, integration, and behavior potentially underestimated.

Our study clearly points toward a reinforcing effect of anodal DCS in a cell-based restorative therapeutic approach in PD. Spontaneous motor behaviors should be investigated in the future in addition to drug-induced behaviors and for a longer period of time after transplantation to better dissect DCS-specific behavioral aspects. Moreover, it is tempting to specifically explore the role of neurotrophins, particularly BDNF, as a mechanistic key candidate for the promotion of graft-induced functional recovery by anodal DCS *in vivo*. Since cell-based therapeutic approaches in primates include bilateral grafting it may be also interesting to investigate the effects of bilateral anodal DCS in this scenario. Given that anodal DCS is safe, well tolerated, established in clinical settings and strategies to handle trepanation-related safety issues are already defined, our results provide a strong basis for future translational research in stem cell-based restorative therapy.

## Synthesis

Reviewing Editor: Julie Andersen, Buck Institute Decisions are customarily a result of the Reviewing Editor and the peer reviewers coming together and discussing their recommendations until a consensus is reached. When revisions are invited, a fact-based synthesis statement explaining their decision and outlining what is needed to prepare a revision will be listed below. The following reviewer(s) agreed to reveal their identity: Manuj Ahuja, Hitoshi Inokawa.

While the reviewers and myself all view your work as an advance to the field, there are several issues that need to be addressed. If you feel that you are able to respond to the reviewers' comment, we would be happy to consider a resubmission of this work. Please see specific comments below:

Reviewer #1:

The current study establishes the beneficial effects of a-tDCS (anodal transcranial direct current stimulation) on the survival and integration of fetal dopaminergic transplants in oxidopamine based hemiparkinsonian PD model. The study provides sufficient evidence to show for the first time that a-tDCS can help in better sustaining the cell transplants in the PD animal model. It will open the new avenue where investigators can advance in developing a better approach to treat PD. The study is well designed and the results are discussed appropriately. As it is the proof of concept study, the authors have established very well that a-tDCS is an effective approach to improve cell transplants therapy in the pre-clinical setting. However, there is lot more to be covered experimentally to understand the mechanism and improve its employability in the clinical setting.

1. For this experiment as reported by authors in line 110 on page 6, female Sprague-Dawley rats were used. The reviewer wants to know why only female rats were used for this study? Gender influences the 6-OHDA induced dopaminergic cell loss and female rats have shown to have less dopaminergic cell loss after 6-OHDA challenge.

2. This reviewer feels that the figures can still be improved by providing more clear images in Figure 3D.

3. Clearly this is the proof of concept study, however the authors could have further determined the mechanism underlying the ability of a-tDCS in facilitating transplanted dopaminergic cell survival and integration.

Reviewer #2:

The authors proposed that cell implantation with direct current stimulation (DCS) promote survival rate of the cells. The concept is interesting and success of this new methodology will advance cell transplantation therapy. However, I think that the evidence provided is still weak even though this is a proof-of-concept study for reasons given below.

Major:

Increased BDNF content by DCS is an important result, however the effect of DCS on the production of BDNF protein is not clear. The authors state that the relative BDNF protein difference between DCS and sham control is statistically significant (p value of 0.049). However from the value of mean and standard error of mean described in the text (lines 287 -289), this does not appear to be the case. Using the values given in the text, the calculated p value would be:

(Used this linkhttp://graphpad.com/quickcalcs/ttest1/?Format=SEM)

P value and statistical significance:

The two-tailed P value equals 0.0698

By conventional criteria, this difference is considered to be statistically significant.

Confidence interval:

The mean of DCS minus Sham equals 21.000

95% confidence interval of this difference: From -1.946 to 43.946

Intermediate values used in calculations:

t = 1.9629

df = 14

standard error of difference = 10.699

Error bars in the figure 2 between sham and anoda also appear to be incorrect. This puts into question proof of the essential concept of this current study.

The authors also emphasize their behavioral recovery results, but these do not seem to unequivocally support their claim. Results from ANOVA show significance in the factor TIME not in Group. In addition, five weeks after the transplantation, behavior was clearly recovered not only in DCS but also in sham treated animals. The authors also state that the number of fully recovered animals was largest in anodal DCS (67%), but when comparing the number of cases of fully recovered animals amongst the groups by Fisher's exact test, there was no significant differences between the groups. Thus, this result does not support the author's hypothesis.

The authors claim that the anodal DCS significantly enhanced graft survival and dopaminergic re-innervation of the striatal tissue relative to sham although the number of TH-ir cells were not different between DCS and sham. This could mean that dopaminergic cells may have longer axons an/or more complex axon collaterals compared to sham. At present,the mechanism underlying this phenomenon is unclear, although important in improving future cell transplantation therapy.

Minor:

The manuscript has many typos and incomplete descriptions.

Line 293 13.000 ->130,000

Line 300 41{plus minus}190 cells, n=7; (Z -> t they used t-test.

Line 302 t(14) = 1-01 -> -1.01?

Line 309 DCS (n=7, t(14)=-2.54, p=0.024; Figures 3D, 4A) Mean and SED were not described

Line 313 T=-0.606 t = -0.606

Line 315 sham DCS n=7: t(14)=-2.67, Mean and SED were not described

Line 335 0.87{plus minus}0.19 mm3, n=7; t(14) -> t(13)

Line 342 volume: t(13)=-0.94, p=0.37 ; Figure 4A, B; density: t(13)=0.1, p=0.92). Mean and SED were not described

Line 345-6 statistically there was a strong trend (t(13) =-2.02, p=0.07... may be wrong value. 0.07 is not strong trend

Line 413 TH-ir cells per total graft core volume were similar... They described “Higher” in results section (P13).

Figure 3D is poor resolution.
